# Editorial: IgE and its receptors in the context of allergy

**DOI:** 10.3389/falgy.2024.1471097

**Published:** 2024-08-28

**Authors:** Paul Engeroff, Sergio Villazala-Merino

**Affiliations:** ^1^ Department of Rheumatology and Immunology, University Hospital Bern, Bern, Switzerland; ^2^ Department for BioMedical Research, University of Bern, Bern, Switzerland; ^3^ Centre D'Immunologie de Marseille-Luminy (CIML), Aix Marseille Université, INSERM, CNRS, Marseille, France

**Keywords:** FcɛRII (CD23), FcɛRI, atopy, B cells, mast cells, basophils

**Editorial on the Research Topic**
IgE and its receptors in the context of allergy

IgE-mediated allergy, also known as Type I hypersensitivity, occurs when the immune system overreacts to a typically harmless substance known as an allergen. Firstly and in response to an allergen encounter, allergen-specific IgE is produced by B cells (1). Then, allergen-specific IgE quickly binds to IgE receptors, primarily the high-affinity receptor FcɛRI and the low-affinity receptor FcɛRII (CD23). These receptors play pivotal roles in initiating and regulating the allergic reaction. FcɛRI is found on mast cells and basophils and is responsible for triggering immediate hypersensitivity reactions when allergens cross-link FcɛRI-bound allergen-specific IgE, leading to degranulation and release of inflammatory mediators (2). The basophil activation test (BAT), which measures IgE-dependent FcɛRI activation and effector cell degranulation in response to allergen challenge, is used as a diagnostic and monitoring tool for allergic disease (3). CD23, which is constitutively expressed on B cells, but can be up-regulated in a variety of cell types, regulates IgE serum levels and participates in secondary immune responses by perpetuating allergen-specific T cell responses (4–6). Besides FcɛRI and CD23, soluble IgE receptors and natural anti-IgE autoantibodies are important regulators of IgE and IgE receptor function (7–9). Recent findings have shown that IgE, the most heavily glycosylated antibody, regulates its interaction with IgE receptors and function through said glycosylations (10). A better understanding of IgE biology and its complex interplay with IgE receptors may advance our ability to treat allergy with IgE-modulating therapies and anti-IgE biologics (11, 12).

The present research topic “*IgE and its receptors in the context of allergy*” includes two review articles covering how allergen (Fröhlich-Nowoisky et al.) and IgE post-translational modifications (Plattner et al.) influence immune responses and IgE-receptor interactions, respectively, as well as original research exploring the diagnostic importance of IgE-FcɛRI interaction (Kunizaki et al. and Akiki et al.) and the modulation of IgE production by a plant flavonoid (Yang et al.). Overall this collection summarizes recent findings or report new original research with the aim of advancing our understanding of IgE and how this immunoglobulin interacts with its receptors in the context of allergy.

A mini-review written by Fröhlich-Nowoisky et al. examines how protein modifications, specifically oligomerization and tyrosine nitration linked to oxidation and exposure to anthropogenic pollutants, affect immune responses to allergens and potentially contribute to the rising prevalence of allergic diseases. This includes the potential effects on B cell activation for specific IgE generation as well as cross-linking of FcɛRI-displayed IgE, triggering an allergic reaction. Following on the importance of post-translational modifications, the review article by Plattner et al. delves into the previously mentioned emerging role of IgE glycosylation in regulating Fc*ɛ*RI vs. CD23 receptor binding and functional consequences. The study discusses how natural anti-IgE autoantibodies may target glycan structure on IgE to modulate its receptor targeting and thus allergic sensitization and inflammation. Additionally, the review discusses the unique structural features flexibility of IgE and its functional complexity.

Additionally, two original research articles use basophil activation test as a diagnostic tool. First, Kunizaki et al. explored whether basophil activation could be linked to exercise-induced allergic reactions on desensitization (EIARDs) in food allergic patients undergoing oral immunotherapy. An exercise provocation test (EPT) was conducted on 20 participants to diagnose EIARD, during which the activation status of basophils was monitored using CD203c and CD63 markers. The findings indicated no significant differences in basophil activation between those who tested positive and those who tested negative for EIARDs during the EPT. This suggests that *in vivo* basophil activation following the ingestion of allergenic foods might not be related to EIARDs, highlighting the need for new diagnostic approaches to predict such reactions. Secondly, Akiki et al. studied the efficacy of using both skin tests and BAT to prevent allergic transfusion reactions (ATRs) in a patient with sickle-cell disease regularly undergoing exchange transfusions. A total of 192 red blood cell (RBC) units were tested, with a high concordance rate of 95% between the two tests. If both tests were negative, the RBC units were transfused; positive results on either test led to unit disposal. Out of 169 RBC units that tested negative on both tests, 118 were safely transfused without any ATRs occurring. The findings suggest that combining BAT and skin tests may effectively prevent ATRs, with skin tests alone also being potential enough. Further research is needed to validate these preliminary results.

Finally, a study by Yang et al. aimed to identify and understand the active compounds in ASHMI (anti-asthma herbal medicine intervention) responsible for suppressing IgE production. The research isolated formononetin from *Sophorae Flavescentis* through various chromatographic and analytical techniques. In tests involving the IgE-producing U266 cell line, formononetin significantly reduced IgE production in a dose-dependent manner without causing cytotoxicity. This reduction was associated with decreased expression of XBP-1 and IgE-heavy chain genes. The findings suggest that formononetin could be a promising candidate for treating allergic asthma and other IgE-mediated diseases.

In summary, this research topic delivered novel insights into the role of IgE and its receptors in the context of allergy. The two review articles give an overview on how allergen as well as IgE post-translational modifications determine IgE-dependent allergic responses and the intricated interplay regulating IgE-Fcɛ receptor interaction. Besides the reviews, two studies using BAT as a diagnostic tool for uncommon allergic reactions and another one investigating a plant compound displaying promising inhibitory capacity of IgE production complete the article collection of this research topic.

These articles collectively enhance our understanding of allergy mechanisms and diagnostic tools, paving the way for more effective management and treatment strategies. The integration of molecular biology with clinical practice in these studies underscores the ongoing evolution in the field of allergology, highlighting the importance of continued research and innovation.
